# Acupuncture with different acupoint combinations for chemotherapy-induced nausea and vomiting: study protocol for a randomized controlled trial

**DOI:** 10.1186/s12906-016-1425-1

**Published:** 2016-11-08

**Authors:** Lili Gao, Bo Chen, Qiwen Zhang, Tianyi Zhao, Bo Li, Tao Sha, Jinxin Zou, Yongming Guo, Xingfang Pan, Yi Guo

**Affiliations:** 1 College of Acupuncture and Massage, Tianjin University of Traditional Chinese Medicine, No. 312, Anshan West Road, Nankai District, Tianjin, 300193 China; 2 Acupuncture Research Center, Tianjin University of Traditional Chinese Medicine, No. 312, Anshan West Road, Nankai District, Tianjin, 300193 China

**Keywords:** Acupuncture, Chemotherapy-induced nausea and vomiting, Acupoint combination, Randomized controlled trial

## Abstract

**Background:**

Acupuncture is beneficial for controlling chemotherapy-induced nausea and vomiting (CINV). However, the effect of different acupoint combinations on controlling CINV remains unknown. This study aims to compare the effects of distal-proximal point association and local distribution point association on controlling CINV.

**Methods/design:**

The study is a single-center, randomized controlled trial. A total of 240 participants will be randomly divided into four groups. The control group will receive standard antiemetic only, whereas three acupuncture groups will receive four electro-acupuncture treatments once a day with the standard antiemetic. Acupuncture group I and II will receive distal-proximal point association (“Neiguan (PC6) and Zhongwan (CV12)”, and “Zusanli (ST36) and CV12”, respectively); Acupuncture group III will receive local distribution point association (“Shangwan (CV13) and CV12”). The primary outcome measures are the frequency and distress of nausea and vomiting. The secondary outcome measures are the grade of constipation and diarrhea, electrogastrogram, quality of life, etc. Assessment is scheduled from the day before chemotherapy to the fifth day of chemotherapy. Follow-ups are performed from the sixth day to the twenty-first day of chemotherapy.

**Discussion:**

Results of this trial will help in evaluating the efficacy and safety of electro-acupuncture with different acupoint combinations in the management of CINV.

**Trial registration:**

ClinicalTrials.gov identifier: NCT02478047.

## Background

Chemotherapy-induced nausea and vomiting (CINV) are common side-effects of many antineoplastic regimens and can occur for several days after treatment [1]. CINV significantly impacts the patient’ quality of life and nutritional status, and may lead to dose reduction or treatment discontinuation, subsequently increasing the risk of disease progression [2]. Although effective guidelines for CINV prevention exist for both moderately and highly emetogenic chemotherapies, adherence to these guidelines is not widely practiced because of their expensive costs and side-effects, such as headaches, dizziness, constipation, and insomnia [1].

Acupuncture treatment is one of the most sought-after therapeutic modalities in complementary and alternative medicine [3]. It is a safe medical procedure with minimal side effects. Evidence for the therapeutic effects of acupuncture on CINV exists [4]. Electroacupuncture for CINV management has been recommended by the American Society of Clinical Oncology [5]. Based on Traditional Chinese Medicine (TCM), the combination of acupoints can strengthen the essential and comprehensive therapeutic effects of acupuncture [6]. However, no consensus currently exists on the optimal acupoint combination for controlling CINV. For example, Gottschling S [7] found that stimulating PC6 (Neiguan), CV12 (Zhongwan), ST36 (Zusanli) and LI4 (Hegu) may effectively prevent CINV; Shen J [8] concluded that electro-acupuncture on PC6 (Neiguan) and ST36 (Zusanli) is more effective in controlling emesis than antiemetic pharmacotherapy alone. Therefore, we propose a randomized, controlled trial to determine the optimal combination of acupoints for CINV management.

In this trial, we aim to determine whether different acupoint combinations similarly manage CINV. We also aim to determine whether distal-proximal point association or local distribution point association more efficiently manages CINV.

## Methods/design

### Objectives

This study aims to: (1) assess the clinical efficacy and safety of distal-proximal point association and local distribution point association by electro-acupuncture for CINV management; (2) assess the patients’ quality of life, anxiety, and depression, as well as other side effects of chemotherapy, such as diarrhea and constipation.

### Hypothesis

According to the theory of TCM, the combination of acupoints can achieve a synergistic effect. In addition, distal-proximal point association and local distribution point association are the classic methods for combinating acupoints. We hypothesize that distal-proximal point association will achieve better therapeutic effect by reducing toxicity and enhance efficacy in CINV management.

### Design

**Fig. 1:**
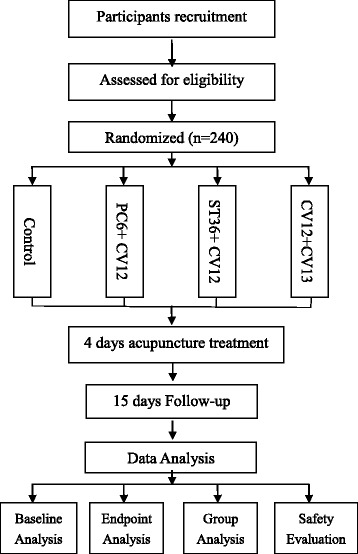
Flow chart of the study

This is a four-armed parallel randomized controlled trial (RCT). 240 participants will be randomly assigned to four groups (a control group and three acupuncture groups) through central randomization in a 1:1:1:1 ratio. The flow chart is shown in Fig. 1.

### Recruitment

All participants will be recruited from the Tianjin Medical University Cancer Institute and Hospital, and the clinical trial information will be posted on notice boards of the hospital. Our trial started on June 2015, and the first participant was recruited on 26 June 2015. For eligible participants who meet all required criteria, it will be requested to sign the written informed consent before randomization, meanwhile they will be given enough time to decide whether they willing to join this study.

### Inclusion and exclusion criteria

#### Inclusion criteria

Participants who meet all of the following criteria will be considered for enrollment. The inclusion criteria are as follows: (1) Diagnosed with cancer and need chemotherapy. (2) The score of Karnofsky (KPS) ≥ 70. (3) Between 18 and 80 years old (either sex). (4) Receiving chemotherapy as outpatients or inpatients. (5) Receiving chemotherapy for one or multiple cycles, but the patient will be taken into the study only once. (6) Patients receiving chemotherapy containing cis-platinum (cis-platinum ≥ 75 mg/m^2^) or antharcycline-combined chemotherapy (doxorubicin ≥ 40 mg/m^2^ or epirubicin ≥ 60 mg/m^2^). (7) Life expectancy ≥ 6 months. (8) Willing to participate in the study and be randomly allocated into one of the four study groups.

#### Exclusion criteria

Participants who meet any of the following will be excluded: (1) Receiving radiotherapy concurrently with chemotherapy. (2) Gastrointestinal tumors. (3) Liver disease with serious complications, or with serious abnormal hepatorenal function (glutamic oxalacetic transaminase (AST), glutamic-pyruvic transaminase (ALT), or total bilirubin (TBIL) three times higher than normal, blood urea nitrogen (BUN) or urine creatinine (Cr) two times higher than normal). (4) Presence of cardiac pacemaker. (5) Active skin infection. (6) Nausea and/or vomiting resulting from opioids or metabolic imbalances, such as electrolyte disturbances. (7) Unable to provide self-care or communicate, or have mental illness. (8) Nausea and/or vomiting resulting from mechanical risk factors, such as, intestinal obstructions. (9) Brain metastases or intracranial hypertension. (10) Pregnant or lactating.

### Randomization

After signing an informed consent and evaluating the inclusion and exclusion criteria, participants will be randomly assigned to one of four groups by center randomization. The central randomized system will be used and performed by the Clinical Evaluation Center at China Academy of Chinese Medical Science in Beijing. A random number and group assignment will be immediately obtained through the website at http://118.144.35.11/crivrs/index.htm. Then the practitioner will assign the participant to that intervention and the evaluator will perform a baseline evaluation.

### Blinding

The evaluator and the statistician are blinded to the intervention a participant accepts. Acupuncturists and patients are not blinded to the treatments they deliver because of the nature of the intervention.

### Intervention

There are four arms in this randomized controlled trial. The control group is supplied with standard antiemetic alone, and the other three arms consist of antiemetic drug and acupuncture.

#### Control groups

Participants in the control group received standard antiemetic alone. Standard antiemetic for all groups is based on the American Society of Clinical Oncology Clinical Practice Guideline [9]. 5-hydroxytryptamine-3 (5-HT3) antagonist (Ramosetron, Tropisetron) and dexamethasone will be administered before the chemotherapy treatment.

#### Acupuncture groups

Three acupuncture groups will receive electro-acupuncture with different acupoint combinations: Acupuncture group I and II will receive distal-proximal point association, and Acupuncture group III will receive local distribution point association. The acupuncturists are the members who hold a Chinese medicine practitioner license from the Ministry of Health of the People’s Republic of China.

##### Acupuncture group I: acupoints “PC6+ CV12” plus antiemetic drug

Participants in Acupuncture group I will receive electro-acupuncture at the Neiguan (PC6) point and Zhongwan (CV12) point. The location of Neiguan (PC6) is on the anterior aspect of the forearm, between the tendons of the palmaris longus and the flexor carpi radialis, 2 B-cun proximal to the palmar wrist crease [10]. The location of Zhongwan (CV12) is on the upper abdomen, 4 B-cun superior to the center of the umbilicus, on the anterior median line [10].

##### Acupuncture group II: acupoints “ST36+ CV12” plus antiemetic drug

Participants in Acupuncture group II will receive electroacupunture at Zusanli (ST36) and Zhongwan (CV12) points. The location of Zusanli (ST36) is on anterior aspect of the leg, on the line connecting ST35 with ST41, 3 B-cun inferior to ST35 [10]. The location of Zhongwan (CV12) has been introduced above.

##### Acupuncture group III: acupoints “CV12+ CV13” plus antiemetic drug

Participants in Acupuncture group III will receive electroacupuncture at Zhongwan (CV12) point and Shangwan (CV13) points. The location of Shangwan (CV13) is on the upper abdomen, 5 B-cun superior to the center of the umbilicus, on the anterior median line [10]. The location of Zhongwan (CV12) has been introduced above.

Disposable, stainless steel acupuncture needles will be used in this trial. The acupuncturists will first insert needles into the acupoints, then the needles will be manipulated until“de qi”sensation is achieved. Next, the needle will be connected to an electroacupuncture apparatus. The positive pole is linked to the needle, and the reference pole is secured approximately one cm in the proximity of the acupoint with a plaster. The electro-acupuncture treatment will use a frequency of 2/10 Hz, and the intensity of stimulation will be adjusted according to the patient’s tolerance. The electric current will be set at less than 10 mA. The procedure will last for 30 min. Treatment is scheduled to occur 30–60 min before chemotherapy infusion and will last for 4 days. Detailed information on acupuncture treatment is summarized based on the revised STRICTA recommendations [11] in Table 1.

**Table 1:** Acupuncture treatment details based on the STRICTA checklist [11]

Item	Detail
1.Acupuncture rationale	1a) Style of acupuncture
	- electro-acupuncture
	1b) Reasoning for treatment provided, based on historical context, literature sources, and/or consensus methods, with references where appropriate
	- electro-acupuncture treatment based on the theory of TCM, literature sources, and clinical experience in acupuncture and CINV, such as references [16] and [17].
	1c) Extent to which treatment was varied
	- The treatment was not varied.
2. Details of needling	2a) Number of needle insertions per subject per session (mean and range where relevant)
	- From 2 to 3.
	2b) Names (or location if no standard name) of points used (uni/bilateral)
	- Four points used: ST36 (bilateral); PC6 (bilateral) ;CV12(unilateral); CV13 (unilateral).
	2c) Depth of insertion, based on a specified unit of measurement, or on a particular tissue level
	- PC6: 0.5 body-inches; ST36: 1-1.5 body-inches; CV12 and CV13: 1-1.5 body-inches.
	2d) Response sought (e.g., de qi or muscle twitch response)
	- ‘De qi’ sensation will be achieved by lifting and thrusting combined with twirling and rotating the needles.
	2e) Needle stimulation (e.g., manual, electrical)
	- Electrical stimulation: the frequency is 2/10 Hz, and the intensity of stimulation is adjusted according to the patient’s tolerance (maximum of 10 mA).
	2f) Needle retention time
	- Thirty minutes
	2g) Needle type (diameter, length, and manufacturer or material)
	- A disposable stainless steel acupuncture needle, 0.25mm × 40 mm (Huatuo, Suzhou Medical Co. Ltd., Jiangsu, China).
3.Treatment regimen	3a) Number of treatment sessions
	- Four treatment sessions in acupuncture groups.
	3b) Frequency and duration of treatment sessions
	- Once daily for 4 days, 30 minutes for each session.
4. Other components of treatment	4a) Details of other interventions administered to the acupuncture group (e.g., moxibustion, cupping, herbs, exercises, lifestyle advice)
	- No other interventions.
	4b) Setting and context of treatment, including instructions to practitioners, and information and explanations to patients
	- University hospitals. - Participants will be informed about acupuncture treatment in the study as follows: “In this study, eletroacupuncture for CINV will be used based on traditional Chinese medicine.”
5.Practitioner background	5) Description of participating acupuncturists (qualification or professional affiliation, years in acupuncture practice, other relevant experience)
	- The participating acupuncturists have all majored in acupuncture, have an acupuncture degree, and are qualified doctors of Tradition Chinese Medicine. All have at least 3 years of experience, and will have been trained in the standard operating procedure of electro-acupuncture on CINV. Thus, they are able to provide identical acupuncture treatment in accordance with a pre-defined protocol.
6. Control or comparator interventions	6a) Rationale for the control or comparator in the context of the research question, with sources that justify this choice
	- The control group is supplied with standard antiemetic alone, so as to provide patients with conventional treatment for CINV.
	6b) Precise description of the control or comparator. If sham acupuncture or any other type of acupuncture-like control is used, provide details as for items 1 to 3 above.
	- Participants in the control group will not receive acupuncture treatment.

### Participant timeline

Enrollment will be conducted the day before chemotherapy (day 0). The electro -acupuncture intervention will be given once daily from day 1 to day 4. The schedule of enrollment, interventions, and assessments is shown in Table 2.

**Table 2:** The schedule of enrollment, interventions, and assessments

	Study period								
	Enrolment	Allocation	Post-allocation						Close-out
Time points	-t1	t0	t1	t2	t3	t4	t5	t6-t20	t21
Enrollment									
Eligibility screen	√								
Informed consent	√								
Allocation		√							
Interventions									
Control group									
Experimental group I			√	√	√	√			
Experimental group II			√	√	√	√			
Experimental group III			√	√	√	√			
Assessments									
Baseline variables	√	√							
The frequency of nausea and vomiting		√	√	√	√	√	√	√	√
The grading of nausea and vomiting		√	√	√	√	√	√	√	√
Rhodes Index of nausea, vomiting and retching		√	√	√	√	√	√	√	√
The grading of constipation and diarrhea		√					√		√
eletrogastrogram		√					√		√
quality of life		√					√		√
anxiety and depression		√					√		√
Other adverse effect during the chemotherapy							√		√
The adverse effects of acupuncture			√	√	√	√			

t0, the day before chemotherapy; t1-t5, the first day to the fifth day of chemotherapy; t6-t21, the sixth day to the 21st day of chemotherapy

### Outcome measures

The observation period will cover the day before chemotherapy (day 0) to the fifth day of chemotherapy (day 5). Follow-ups will be conducted from the sixth day (day 6) to the twenty first day (day 21) of chemotherapy. Participants will be asked to keep CINV diaries from the sixth day (day 6) to the twentieth day of chemotherapy (day 20).

#### Primary outcome measures

The primary outcome measures are as follows:

1. Frequency of nausea and vomiting: total nausea and vomiting episodes per person over the 6-day study period will be recorded. The frequency of nausea and vomiting is the most valuable measurement for evaluation.
2. Grading of nausea and vomiting: nausea and vomiting will be graded by Common Terminology Criteria for Adverse Events Version 4.0 [12].
3. Rhodes Index of Nausea, Vomiting and Retching: nausea, vomiting and retching will be measured as separate entities by Rhodes Index of Nausea, Vomiting and Retching, which have 8 items with 5-point Likert scales. The frequency and distress of all entities are measured, as well as the duration of nausea and the amount of vomitius. [13]

#### Secondary outcome measures

Secondary outcome measures include the following:

1. Grading of constipation and diarrhea: constipation and diarrhea will be evaluated by Common Terminology Criteria for Adverse Events Version 4.0 [12].
2. Electrogastrogram: gastrointestinal motility will be monitored by electrogastrogram.
3. Assessment of quality of life: the Functional Assessment of Cancer Therapy- General (FACT-G) [14] will be applied to assess four domains of patient quality of life, including physical, social/family, emotional, and functional well-being.
4. Assessment of anxiety and depression: the Hospital Anxiety and Depression Scale [15] will be used to assess anxiety with seven items and depression with another seven items.
5. Other adverse effects, including appetite, will be assessed by Common Terminology Criteria for Adverse Events Version 4.0 [12].

### Sample size calculation

A sample size calculation was performed based on the result of a trial by Shen J [8] and the results of a pilot study. To detect a significant difference between any two groups with a power of 90 % and type I error of 5 %, the calculated number of patients is 192. Considering a 20 % drop-out rate, the total sample size needs 240 patients and 60 patients in each group.

### Statistical analysis

Statistical analysis will also be performed by the Clinical Evaluation Center at China Academy of Chinese Medical Science. The statistician is blinded from the allocation of groups. The Kruskal-Wallis test will be employed in the analysis of skewed distribution data. Chi-square analysis will be used for categorical variables, and Analysis of variance (ANOVA) for numerical variables. Repeated measures analysis will be used in the different time point assessment. A *P* value less than 0.05 is regarded as statistically significant.

### Data management

To promote data quality, the data will be collected by well-trained assessor and the double entry of the data will be implemented by clinical research coordinators.

### Safety

Participants will also be monitored for any adverse events, including swelling, pain, bruise at the sites of needle insertion, or discomfort, palpitation, dizziness, etcetera after acupuncture treatment. Adverse effects will be recorded in the case report forms (CRF).

### Quality control

All researchers will be required to undergo special training classes to guarantee the quality of the study. The training classes will introduce researchers to the trial’s details. For example, researchers will be trained to use the central randomized system, to fully understand the inclusion and exclusion criteria, and to fill in the CRFs. Additionally, clinical monitors will also check the trial processes and CRFs. Patient drop-outs and their reasons will be fully recorded.

### Ethics and dissemination

Medical Ethics Committees at Tianjin University of Traditional Chinese Medicine has approved this protocol (approval number is TJUTCM-EC20140006 and). Patients’ data will be stored securely and the results of the present study will be published in a peer-reviewed journal.

## Discussion

This study protocol expands current literature regarding the efficacy of acupuncture on CINV. Previous reviews showed that acupoint stimulation has a certain effect on CINV. However, the effects of different acupoint combinations are inconsistent. We designed our trial to compare the effects of distal-proximal point association and local distribution point association.

More than 300 acupuncture points are located on the body and each point has its own therapeutic indication. The correct selection and combination of acupoints are the keys to the curative effects of acupuncture [6]. Before developing the trial protocol, we screened a range of published articles and ancient books. Our literature search showed that PC6, ST36, and CV12 are the most commonly used acupoints for managing CINV [16, 17]. We selected PC6 and ST36 as the distal acupoints, and CV12 as the local acupoint. Based on the theory of Chinese acupuncture, distal-proximal acupoint association and local distribution point association are the two classic methods for combining acupoints. Distal-proximal acupoint association is also the most commonly used method in clinical applications. In this trial, there are three different acupuncture groups: “PC6 and CV12” and “ST36 and CV12” are the two disal-proximal acupoint association groups, and “CV12 and CV13” is the local distribution point association group. Therefore, we expect that distal-proximal point association (“PC6 and CV12” or “ST36 and CV12”) will have better effect than local distribution point association (“CV12 and CV13”). Furthermore, we can screen the most effective acupoint combination for clinical application from these three groups.

Although the manipulators and participants cannot be blinded to the group allocation in this study, all outcome measures will be administered and collected by a blinded estimator, and statisticians blinded to group assignment will perform data analysis. With respect to clinical quality control, the manipulators, estimators and statisticians will work independently to reduce the adverse impacts of artificial factors on data. A third party has been invited to manage data independently and to design a central randomization system for minimizing bias and enhancing clinical research quality.

In conclusion, the results of this trial are expected to assess the clinical efficacy and safety of distal-proximal point association and the local distribution point association by electro-acupuncture in the management of CINV.

## Trial status

The first participant was recruited on 26 June 2015. This study is currently recruiting participants.

## Abbreviations

ALT: Glutamic-pyruvic transaminase
ANOVA: Analysis of Variance
AST: Glutamic oxalacetic transaminase
BUN: Blood urea nitrogen
CINV: Chemotherapy- induced nausea and vomiting
Cr: Urine creatinine
CRF: Case Report Form
CV12: The acupoint of Zhongwan
CV13: The acupoint of Shangwan
FACT-G: The Functional Assessment of Cancer Therapy - General
KPS: The scoring of Karnofsky
LI4: The acupoint of Hegu
PC6: The acupoint of Neiguan
RCT: Randomized Controlled trial
ST36: The acupoint of Zusanli
STRICTA: Standards for Reporting Interventions in Clinical Trials of Acupuncture
TBIL: Total bilirubin
TCM: Traditional Chinese Medicine

## References

[1] Schwartzberg LS, Modiano MR, Rapoport BL, Chasen MR, Gridelli C, Urban L (2015). Safety and efficacy of rolapitant for prevention of chemotherapy-induced nausea and vomiting after administration of moderately emetogenic chemotherapy or anthracycline and cyclophosphamide regimens in patients with cancer: a randomised, active-controlled, double-blind, phase 3 trial. Lancet Oncol.

[2] Bloechl-Daum B, Deuson RR, Mavros P, Hansen M, Herrstedt J (2006). Delayed nausea and vomiting continue to reduce patients’ quality of life after highly and moderately emetogenic chemotherapy despite antiemetic treatment. J Clin Oncol.

[3] Han G, Ko SJ, Park JW, Kim J, Yeo I, Lee H (2014). Acupuncture for functional dyspepsia: study protocol for a two-center, randomized controlled trial. Trials.

[4] Wu X, Chung VC, Hui EP, Ziea ET, Ng BF, Ho RS (2015). Effectiveness of acupuncture and related therapies for palliative care of cancer: overview of systematic reviews. Sci Rep.

[5] Naeim A, Dy SM, Lorenz KA, Sanati H, Walling A, Asch SM (2008). Evidence-based recommendations for cancer nausea and vomiting. J Clin Oncol.

[6] Zhao JS. Chinese acupuncture and moxibustion. 1st ed. Shanghai: Publishing house of Shanghai University of Traditional Chinese Medicine; 2002.

[7] Gottschling S, Reindl TK, Meyer S, Berrang J, Henze G, Graeber S (2008). Acupuncture to alleviate chemotherapy-induced nausea and vomiting in pediatric oncology-a randomized multicenter crossover pilot trial. Klin Padiatr.

[8] Shen J, Wenger N, Glaspy J, Hays RD, Albert PS, Choi C (2000). Electroacupuncture for control of myeloablative chemotherapy-induced emesis: A randomized controlled trial. JAMA.

[9] Basch E, Prestrud AA, Hesketh PJ, Kris MG, Feyer PC, Somerfield MR (2011). Antiemetics: American Society of Clinical Oncology clinical practice guideline update. J Clin Oncol.

[10] World Health Organization, WHO standard acupuncture point locations in the Western Pacific Region. Manila: World Health Organization Regional Office for the Western Pacific; 2008.

[11] MacPherson H, Altman DG, Hammerschlag R, Li YP, Wu TX, White A (2010). Revised STandards for Reporting Interventions in Clinical Trials of Acupuncture (STRICTA): extending the CONSORT statement. PLoS Med.

[12] US Department of Health and Human Services, Common terminology criteria for adverse events (CTCAE) version 4.0. Bethesda: National Institutes of Health, National Cancer Institute; 2009.

[13] Rhodes VA, McDaniel RW (1999). The index of nausea, vomiting, and retching: a new format of the index of nausea and vomiting. Oncol Nurs Forum.

[14] Fairclough DL, Cella DF (1996). Functional Assessment of Cancer Therapy (FACT-G): non-response to individual questions. Qual Life Res.

[15] Snaith RP (2003). The hospital anxiety and depression scale. Health Qual Life Outcomes.

[16] An Q, Chen B, Guo Y, Pan XF, Guo YM (2015). A preliminary discussion on rules of clinical acupoint selection of acupuncture for the treatment of chemotherapy-induced nausea and vomiting. World J Acupunct Moxibustion.

[17] Bao T (2009). Use of acupuncture in the control of chemotherapy-induced nausea and vomiting. J Natl Compr Canc Netw.

